# Unearthing anti-MRSA agents from alpine lichens: discovery and characterization of bioactive compounds in *Cetraria islandica* from the snowy Cangshan region

**DOI:** 10.3389/fmicb.2025.1688435

**Published:** 2025-11-27

**Authors:** Junlin Lu, Hongqiao Tian, Fangrong Liang, Zhiyi Xiang, Menglong Liu, Haiyan Ding

**Affiliations:** College of Public Health, Dali University, Dali, China

**Keywords:** Cangshan lichen, *Cetraria islandica*, lichesterinic acid, antibacterial activity, toxicity evaluation

## Abstract

Methicillin-resistant *Staphylococcus aureus* (MRSA)—declared a WHO priority pathogen—remains a global menace, yet no new-scaffold agent has reached the clinic in two decades. The under-investigated chemical reservoir of lichens was tapped by targeting *Cetraria islandica* collected in Cangshan, Yunnan. Bioassay-guided fractionation yielded lichesterinic acid (C_19_H_32_O_4_, 95% purity, 0.32% yield), whose structure was elucidated by ^1^H/^13^C NMR and HRESI-MS. Antimicrobial spectrum testing revealed that lichesterinic acid exhibited a minimum inhibitory concentration (MIC) of 64–128 μg/mL against *Staphylococcus aureus*, MRSA, and *Listeria seeligeri* and an inhibitory rate greater than 70% against phytopathogenic fungi such as *Sclerotinia sclerotiorum* and *Valsa mali*. When combined with six different antibiotics, it exhibited synergistic or additive effects, suggesting its potential to restore sensitivity to traditional antibiotics. Cytotoxicity assays of HepG2 and Vero cells showed IC_50_ values of 1,854 and 1,771 μM, respectively. Acute oral toxicity tests in mice revealed no deaths or significant toxicity, with an LD_50_ > 5,000 mg/kg, indicating that the drug was nontoxic. Molecular docking studies revealed that lichesterinic acid may stabilize key resistance proteins such as deacetylase (def) and PBP2a, potentially exerting multitarget antimicrobial effects by inhibiting protein synthesis and cell wall formation. In summary, lichesterinic acid is a safe, low-toxicity, broad-spectrum candidate for a new type of natural antimicrobial agent, providing a material basis and theoretical foundation for the development of MRSA drugs.

## 1 Introduction

Under the continuous pressure of antibiotics, superbugs have become one of the three major public health threats of the 21st century, causing 700,000 deaths globally each year (Romandini et al., 2021). It is predicted that by 2050, antibiotic resistance will lead to a global death toll of 10 million and economic losses of up to $100 trillion (Naghavi et al., 2024). MRSA has spread globally at an alarming rate since it was first reported in 1961, currently ranking alongside hepatitis B and HIV as one of the three most challenging infectious diseases in the world today (Romero and de Souza da Cunha, 2021). MRSA exhibits broad-spectrum resistance not only to β-lactam antibiotics but also to aminoglycosides, tetracyclines, and fluoroquinolones (Wang et al., 2020); only a limited number of antibiotics, most notably vancomycin and linezolid, retain clinical efficacy. Nevertheless, resistance to these “last-line” agents has emerged at varying levels (Lin et al., 2025). Over the past two decades, no novel antibiotic class or target has received regulatory approval, underscoring the urgent need for innovative anti-MRSA therapeutics.

In recent years, as research into natural products has intensified, the search for new bioactive and therapeutic natural compounds derived from microbial metabolites has become a growing trend in drug development. Lichens—unique microbial mutualisms—synthesize an array of distinctive secondary metabolites as an adaptive strategy to cope with the extreme environmental stresses imposed by their specialized habitat and slow-growing lifestyle (Ubek et al., 2021). These metabolites display a broad spectrum of bioactivities—including potent antibacterial, antioxidant, and anti-inflammatory properties—underscoring the exceptional promise of lichens as a reservoir for the discovery of novel antibacterial agents (Do et al., 2022). However, the current understanding of lichens and their active compounds is still relatively inadequate, particularly in terms of their biological activities, which significantly limits the application of lichen resources in the pharmaceutical field. Research teams have previously reported that the crude extract of Cangshan lichen has good antibacterial activity against MRSA and has a broad antibacterial spectrum, indicating the potential for further exploration (Tian et al., 2024). Nonetheless, research on the foundation of antibacterially active substances and the application prospects of Cangshan lichens remains scarce. Therefore, this study employed MRSA as an indicator strain to systematically characterize *C. islandica* by integrating natural product chemistry with pharmacological approaches. Chemical structures of the isolated compounds were elucidated, the *in vitro* antibacterial activity of the bioactive constituent lichesterinic acid was quantitatively determined, and its toxicological profile was established. Furthermore, preliminary insights into the mechanism by which lichesterinic acid acts against MRSA were obtained. These findings provide essential structural and bioactivity evidence to support subsequent pharmacological exploration and the rational development of *C. islandica* as a medicinal resource.

## 2 Materials and methods

### 2.1 Materials and strains

*C. islandica* was collected from the area of Cangshan, Dali, Yunnan Province, China, at an altitude of 3,800–4,100 m (east longitude 100°06′, north latitude 25°36′) and is currently preserved in the Microbiology Research Laboratory of the School of Public Health at Dali University. Information on the test strains is provided in Supplementary Table S1, and the reagent information is listed in Supplementary Table S2.

### 2.2 Isolation and identification of metabolites from lichens against MRSA

The lichen crude extract was subjected to ultrasonic extraction in a 75% methanol solution. The extract was then filtered and concentrated under reduced pressure via a rotary evaporator. The lyophilized crude extract was re-dissolved in water and successively partitioned with petroleum ether and ethyl acetate (1:1, v/v). Each organic layer was separated, concentrated under reduced pressure at 4°C, and the resulting residues were immediately placed in amber vials, sealed, and stored at 4°C in the dark until further use. Preliminary fractionation of the petroleum-ether and ethyl-acetate extracts was performed by silica-gel column chromatography. The petroleum-ether fraction was eluted with a stepwise gradient of petroleum ether–methanol (1:0, 100:1, 90:1, 80:1, 70:1, 60:1, 50:1, 40:1, 30:1, 20:1, 10:1, 8:1, 5:1, 3:1, 1:1, 0:1, v/v) to afford 16 distinct fractions. The ethyl acetate extract was similarly eluted via a dichloromethane–methanol gradient, which also yielded 16 components. The anti-MRSA active components were screened via the broth microdilution method (Jarkhi et al., 2022). The active components were further separated into multiple subcomponents via Sephadex LH-20 gel column chromatography (CHCl^3^–MeOH 1:1). Owing to the limited sample availability, the TLC-direct bioautography method (Sun et al., 2019) was used to perform in situ detection of anti-MRSA activity on thin-layer plates. The MRSA bacterial mixture was diluted to 10^5^ CFU/mL and mixed with MHA culture medium at a 20:1 ratio for homogeneous overlay on TLC plates. After incubation at 37°C for 12–18 h, 0.2% 2,3,5-triphenyl-2H-tetrazolium chloride (TTC) was added to detect antibacterial spots. The target compound was subsequently purified by recrystallization from petroleum ether; the resulting crystals were collected by filtration, concentrated under reduced pressure, and dried in vacuo. Final structural elucidation was performed by the Kunming Institute of Botany, Chinese Academy of Sciences, using ^1^H NMR, ^13^C NMR, and electrospray ionization mass spectrometry (ESI-MS).

### 2.3 Determination of the antibacterial spectrum of the active monomer compounds

The minimum inhibitory concentration (MIC) of the purified monomer against MRSA was determined by the standard broth microdilution method using twofold serial dilutions (Jarkhi et al., 2022). A total of 1.024 mg of lichesterinic acid was weighed and dissolved in 3% DMSO to a final volume of 1.0 mL for testing. Chloramphenicol and ciprofloxacin were used as positive controls, and Mueller–Hinton Broth served as the blank control. In a 96-well microtiter plate, serial doubling dilutions of lichesterinic acid, chloramphenicol and ciprofloxacin were prepared from 512 to 1 μg/mL. The bacterial culture was shaken at 37°C to mid-logarithmic phase (OD_600_ ≈ 0.4), harvested by centrifugation (3,000 g, 10 min, 4°C), and re-suspended in sterile 0.85% (w/v) saline. The suspension was adjusted to the 0.5 McFarland standard (≈ 1.5 × 10^600^ CFU mL^−1^) and then diluted to 1 × 10^5^ CFU mL^−1^ in cation-adjusted Mueller–Hinton broth. A 2 μL aliquot of the suspension (2% v/v, final volume 100 μL) was dispensed to each well, and each concentration was tested in triplicate. After incubation at 37°C for 18 h, 20 μL of 0.5% (w/v) TTC working solution was added to each well, followed by an additional 1 h incubation at 37°C. Wells showing no development of red formazan coloration were considered to have no bacterial growth, and the corresponding drug concentration was defined as the minimum inhibitory concentration (MIC).

The antifungal activity of lichesterinic acid against pathogenic fungi was determined via the hyphal growth rate method (Hairu et al., 2022). Pathogenic fungal strains were first cultured on potato dextrose agar (PDA) plates at 28°C for 7 days in the absence of lichesterinic acid. Agar plugs (6 mm diameter) were then excised from the actively growing colony margin and transferred to the center of fresh PDA plates supplemented with lichesterinic acid at a final concentration of 1.0 μg/mL. These plugs were placed in the center of prepared agar plates. Additionally, fungal plugs were placed in media containing 5 μg/mL Iprodione solution as a positive control, whereas plugs inoculated in agar media containing only sterile PDA served as a negative control. Each treatment comprised three replicate plates. After treatment, the plates were inverted and incubated at 28°C for 7 days to observe colony growth. The diameter of the colonies was measured via the cross method, and the inhibition rate was calculated via the following formula (Muhorakeye et al., 2024):


$$\mathrm{Inhibition}\,\mathrm{rate}\%=\frac{\begin{array}{c} \mathrm{Control}\,\mathrm{group}\,\mathrm{colony}\,\mathrm{diameter}-\\ \mathrm{Confrontation}\,\mathrm{culture}\,\mathrm{colony}\,\mathrm{diameter} \end{array}}{\mathrm{Control}\,\mathrm{group}\,\mathrm{colony}\,\mathrm{diameter}}\times 100$$

### 2.4 Evaluation of the combined effects of active monomer compounds and antibiotics

Following the protocol established by Jarkhi et al. (2022), a checkerboard broth-microdilution assay was performed to evaluate the combined antimicrobial activity of lichesterinic acid with selected antibiotics against MRSA and *L. seeligeri*. Serial two-fold dilutions were prepared for each agent on the basis of their individual MICs, yielding final concentrations of 2, 1, 0.5, 0.25, 0.125, 0.0625, and 0.03125 × MIC in a 7 × 7 matrix. Bacterial inoculum in broth was included as a negative growth control, and uninoculated broth served as a sterility blank. Each concentration was tested in three replicate wells. After incubation at 37°C for 18 h, the MICs of the combinations were recorded, and the fractional inhibitory concentration index (FICI) was calculated according to the equation:


$$\mathrm{FICI}=\frac{{\mathrm{MIC}}_{A\,\mathrm{in}\,\mathrm{combo}}}{{\mathrm{MIC}}_{A\,\mathrm{alone}}}+\frac{{\mathrm{MIC}}_{B\,\mathrm{in}\,\mathrm{combo}}}{{\mathrm{MIC}}_{B\,\mathrm{alone}}}$$

The criteria for interpretation were as follows: FICI ≤ 0.5 indicates synergism; 0.5 < FICI ≤ 1 indicates an additive effect; 1 < FICI ≤ 2 indicates no interaction; and FICI > 2 indicates antagonism.

### 2.5 Safety evaluation of the active monomer compounds

A CCK-8 kit was used to evaluate the *in vitro* cytotoxicity of lichesterinic acid against human liver cancer cell lines (HepG2) and African green monkey kidney cell lines (Vero). The cells were cultured in DMEM supplemented with 10% fetal bovine serum (FBS), and the cell concentration in the logarithmic growth phase was adjusted to 5 × 10^4^ cells/mL. A total of 100 μL of cell suspension was added to each well of a 96-well culture plate and incubated at 37°C under 5% CO^2^ for 24 h until a monolayer of cells formed. The supernatant was then discarded, and the cells were washed with PBS. Lichesterinic acid treatment solutions were added to each well at final concentrations of 64, 128, 256, 512, and 1,024 μg/mL (corresponding to 1/8–1/2 × MIC), with three replicates for each concentration, each containing 100 μL. Incubation was then extended for an additional 48 h under identical conditions. Following incubation, the supernatant was aspirated, and the cells were gently washed twice with PBS. Subsequently, 10 μL of CCK-8 reagent mixed with 90 μL of serum-free medium was added to each well, and the plates were incubated for an additional 30 min at 37°C under 5% CO^2^. The optical density (OD) values of each well were measured at a wavelength of 450 nm via a microplate reader to calculate cell viability (Wang et al., 2021). The cell viability was calculated as follows: cell viability (%) = (OD _*treatment*_−OD _*blank*_)/(OD _*control*_−OD _*blank*_) × 100 (Yoon et al., 2018). The blank group consisted of culture medium without cells, the sample group contained cells treated with lichesterinic acid, and the control group consisted of nondrug-treated cell cultures. The experiment was repeated three times to ensure the accuracy and reproducibility of the results.

The acute oral toxicity test was conducted according to the guidelines established by the Organization for Economic Cooperation and Development (OECD) 423 (OECD, 2002) and was approved by the Medical Ethics Committee of Dali University. Specific-pathogen-free C57BL/6J mice (*n* = equal numbers of males and females; 9–12 weeks old; 25.16 ± 3.09 g body weight) were housed under controlled conditions (22–25°C, 50–60% relative humidity, 12 h light/dark cycle) with *ad libitum* access to standard chow and water. Following a 16 h fast (water provided *ad libitum*), animals were randomly assigned to receive a single oral gavage of the test compound or vehicle. Based on the reported LD_50_ of usnic acid (388 mg/kg) (Cheng et al., 2009), five gradient doses were designed (194, 388, 776, 1,552, and 3,104 mg/kg). In the initial phase of the preliminary experiment, three mice were gavaged at doses of 194 and 3,104 mg/kg, and no abnormalities were observed over 14 days. Subsequently, another group of three mice receiving the 3,104 mg/kg dose continued with a higher dose of 5,000 mg/kg lichesterinic acid via gavage, and their food intake, water consumption, and general behavior were observed over 14 days. The results indicated no deaths or signs of toxicity, and their behavior remained normal. For the formal experiment, 20 C57BL/6J mice (equal numbers of males and females, aged 9–12 weeks) were gavaged with a maximum dose of 5,000 mg/kg body weight, whereas the control group (*n* = 5) received an equivalent amount of corn oil. Animals were monitored continuously during the first 4 h post-administration and then at regular intervals for the remainder of the initial 24 h, followed by daily observations for a total of 14 days. Body weights were recorded on days 7 and 14, and daily measurements of food and water intake were obtained. Clinical signs were systematically evaluated and included alterations in respiratory rate, piloerection, stereotypic behaviors, fine tremors, raised forelimbs, convulsions, forward-leaning posture, lowered hindquarters, photophobia, faucal output, abdominal distension, and mortality. In the present study, retro-orbital bleeding was selected as the sampling method (Liu et al., 2022). Mice were rendered deeply anesthetized via isoflurane delivered in 100% oxygen (induction 4–5%; maintenance 1.5–2% v/v) at a flow rate of 0.8–1.0 L min^–1^. Peripheral blood was collected by retro-orbital puncture using sterile, smooth-tipped capillary glass tubes. Following sampling, animals were humanely euthanized and a full necropsy was performed to assess gross organ integrity. All procedures were carried out by trained personnel in accordance with institutional standard operating procedures and approved by the Institutional Animal Care and Use Committee.

### 2.6 Molecular docking of active monomeric compounds and *S. aureus* resistance proteins

To identify the potential targets of lichesterinic acid against MRSA, reverse pharmacophore mapping was performed with the cloud-based PharmMapper platform^1^ (Wang et al., 2017). This tool conducts high-throughput screening of three-dimensional structures hosted in the RCSB protein structure database.^2^ After conversion to PDB format, lichesterinic acid was submitted with all other parameters left at their default values; PharmMapper automatically removed ligands/cofactors from the protein and performed reverse docking. Targets exhibiting the highest similarity scores and the closest functional association with MRSA pathogenesis were selected; these were subsequently subjected to high-precision molecular docking, molecular-dynamics simulation (MD), and structure–activity relationship (SAR) analyses were conducted on these targets.

To evaluate the binding affinity between lichesterinic acid and the target identified by reverse virtual screening, molecular docking simulations were employed for validation (Yang et al., 2023). The structure of the target ligand lichesterinic acid was retrieved from the ChemPub database, and the X-ray crystal structure of the target protein ClpP was sourced from the RCSB protein database. To ensure that the protein structure was suitable for molecular docking experiments, PyMOL software was employed for preprocessing, which involved removing water molecules, ligands, and irrelevant cofactors and adding polar hydrogen atoms. Subsequently, AutoDock Tools software was used for conformational optimization and preprocessing of lichesterinic acid, preparing it for docking analysis with the protein. The processed molecule was utilized as a ligand, and molecular docking experiments were conducted with AutoDock Vina software to evaluate its affinity by calculating the binding energy between the ligand and the receptor. The conformation with the lowest binding energy was ultimately selected as the optimal binding model for subsequent analyses (Liu et al., 2021). The docking results were visually displayed in three dimensions via PyMOL and Discovery Studio software to facilitate a more intuitive analysis of the interaction features between the ligand and the target.

### 2.7 Data processing

The data were processed and analyzed via IBM SPSS Statistics 27.0 software, with statistical methods including variance analysis and *t*-tests; *p* < 0.05 indicated a significant difference. Single-factor variance analysis and independent sample *t*-tests were employed for comparisons among groups, followed by Dunnett’s multiple comparison test for significance evaluation, where *p* < 0.05 signifies a statistically significant difference. Bar charts and error line graphs were plotted via GraphPad Prism 9.5, whereas scatter plots and regression curves were created via Origin 2021.

## 3 Results

### 3.1 Isolation and identification of antimicrobial metabolites against MRSA from *C. islandica*

**FIGURE 1:**
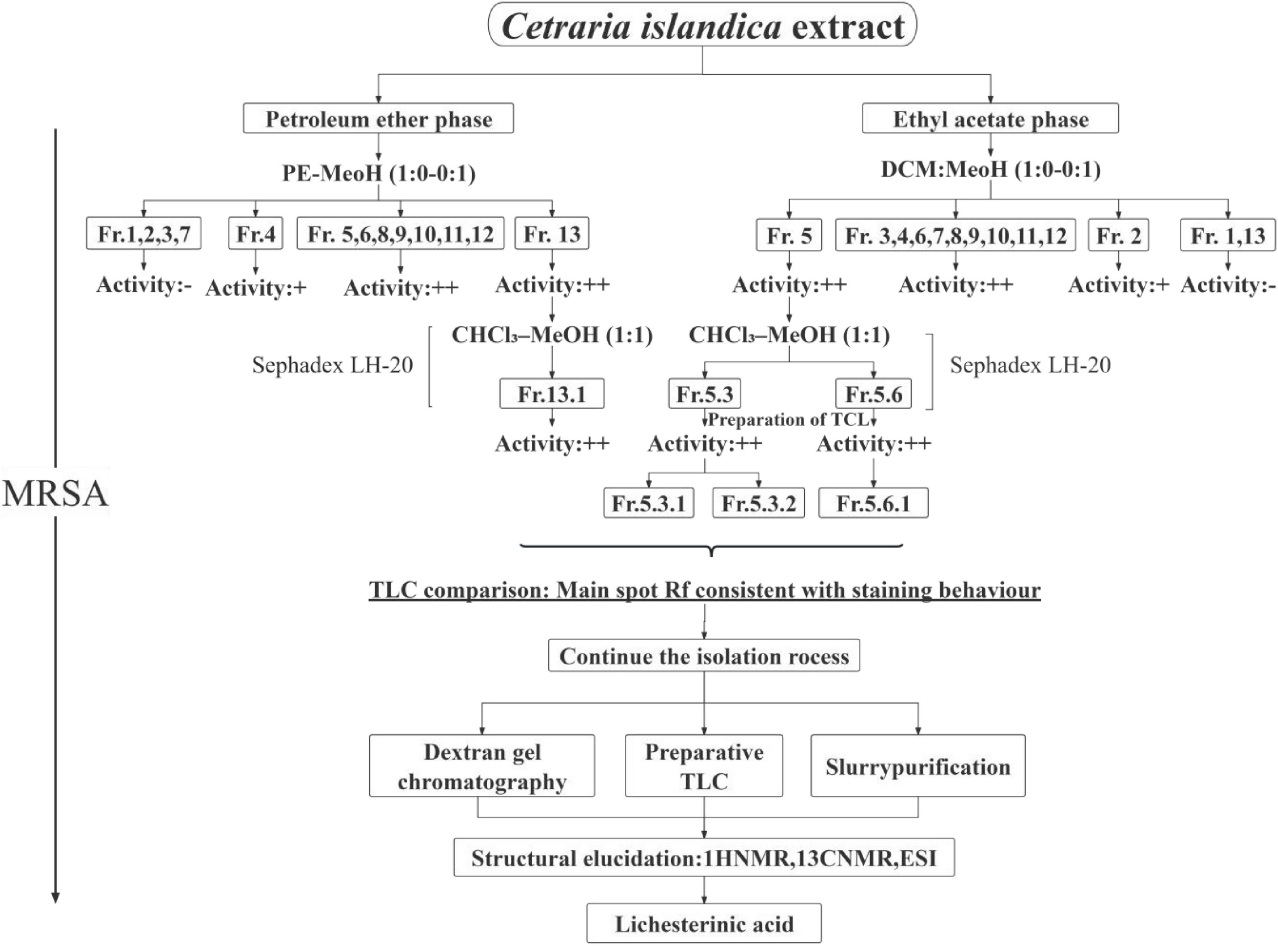
Isolation and purification workflow of metabolites from *C. islandica.* The schematic illustrates the sequential procedure used for the extraction and purification. “+” Indicates moderate anti-MRSA activity (bacterial growth partially suppressed; broth shows slight red coloration with TTC). “++” Indicates strong anti-MRSA activity (complete inhibition; broth remains colorless with TTC).

**FIGURE 2:**
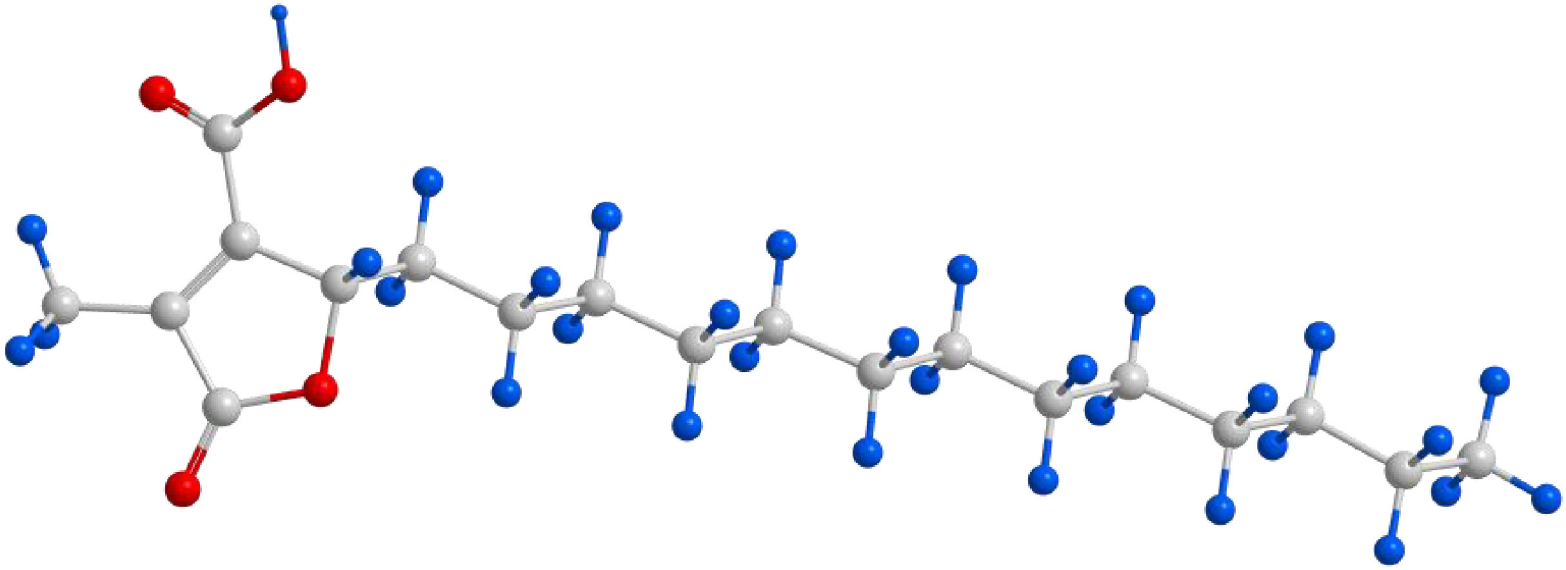
Structural formula of lichesterinic acid. The compound is a bioactive secondary metabolite isolated from *C. islandica*.

After separation and purification through silica gel column chromatography, gel filtration chromatography, and thin-layer chromatography (TLC), a monomeric compound was successfully isolated from the lichen extract (Figure 1). Through analyses via proton nuclear magnetic resonance (^1^H NMR), carbon nuclear magnetic resonance (^13^C NMR), and high-resolution electrospray ionization mass spectrometry (HRESI-MS), this compound was identified as lichesterinic acid (CAS: 493-47-0) (Figure 2). A total of 9.53 g of this white powder product was obtained, corresponding to a dry weight yield of 0.32% and approximately 95% purity. Under a 254 nm UV lamp, TLC indicated that the compound appeared as a single spot, which did not show color when it was sprayed with a 5% ethanol solution of sulfuric acid and heated, suggesting that it may lack common chromogenic groups such as phenolic hydroxyls. High-resolution electrospray ionization mass spectrometry (HRESI-MS) was used to determine the molecular formula of the compound as C_19_H_32_O_4_, with a molecular weight of 324.455. ^1^H-NMR (ppm): 2.50 (d, 3H, H-3), 4.66–4.69 (m, 1H, H-6), 1.17–1.37 (m, ^1^H, H-7a), 2.04 (m, 1H, H-7b), 1.23 (m, 22H, H8–18), 0.83 (t, 3H, H-19). ^13^C-NMR (ppm): 171.3 (C1), 135.5 (C2), 10.89 (C3), 168.7 (C4), 149.0 (C5), 81.88 (C6), 31.88 (C7), 24.90 (C8), 29.6–31.88 (C9–17), 22.67 (C18), 14.49 (C19). The measured ^1^H NMR, ^13^C NMR, and mass spectrometry data were highly consistent with the spectral data of lichesterinic acid reported in the literature, further confirming the structure of the compound (Ogbaji Igoli et al., 2014).

### 3.2 Antibacterial activity of lichesterinic acid against pathogenic bacteria and plant fungi

*In vitro* antibacterial activity tests demonstrated that lichesterinic acid exhibited varying degrees of antibacterial efficacy against 16 strains of pathogenic bacteria (Table 1). The MIC values ranged from 64 to 512 μg/mL. Among all strains, it showed the strongest antibacterial activity against *S. aureus* and *L. seeligeri*, with MIC values of 64 μg/mL for both, where the activity against the latter was comparable to that of ciprofloxacin. Lichesterinic acid also demonstrated inhibitory effects against MRSA, *Salmonella Paratyphi* A and B, and *Pseudomonas fluorescens*, with an MIC value of 128 μg/mL. Additionally, the MIC for *Staphylococcus epidermidis* and *Pectobacterium carotovorum* was 256 μg/mL, while its inhibitory effects on other bacteria were relatively weak.

**TABLE 1:** Minimum inhibitory concentration of lichesterinic acid against pathogenic bacteria.

Classification	Strain	MIC (μg/mL)		
		Lichesterinic acid	Chloramphenicol	Ciprofloxacin
G+	MRSA	128	128	64
	*Staphylococcus aureus*	64	16	16
	*Staphylococcus epidermidis*	256	8	8
	*Rhodococcus equi*	512	16	32
	*Listeria seeligeri*	64	8	64
	*Listeria ivanovii*	512	16	32
	*Listeria monocytogenes*	512	16	16
	*Listeria innocua*	512	16	2
G−	*Klebsiella pneumoniae*	512	8	4
	*Salmonella* spp.	512	8	2
	*Salmonella paratyphi A*	128	8	8
	*Salmonella paratyphi B*	128	16	< 1
	*Pseudomonas fluorescens*	128	8	8
	*Pectobacterium carotovorum*	256	16	8
	*Escherichia coli*	512	8	8
	*Shigella flexneri*	512	4	4

The inhibitory effects of lichesterinic acid on nine fungi are presented in Table 2. The results indicate that this compound significantly inhibited the *S. sclerotiorum* and *V. mali*, with inhibition rates exceeding 70% for both. Notably, the inhibitory effect of lichesterinic acid on *V. mali* was comparable to that of the positive control, iprodione. Lichesterinic acid also demonstrated an inhibition rate of over 60% against *Botrytis cinerea* and *Colletotrichum orbiculare*, indicating its potential as an antifungal agent against plant pathogenic fungi.

**TABLE 2:** Evaluation of the activity of lichesterinic acid against plant-pathogenic fungi.

Strain	Inhibition zone diameter(%)	
	Lichesterinic acid	Iprodione
*Colletotrichum orbiculare*	62.7 ± 0.1*^Bab^*	68.5 ± 0.1*^Ab^*
*Fusarium moniliforme*	30.4 ± 0.1*^Bc^*	31.8 ± 0.1*^Ad^*
*Fusarium graminearum*	29.2 ± 0.1*^Bc^*	44.3 ± 0.1*^Ac^*
*Fusarium oxysporum* f. sp*.niveum*	37.1 ± 0.1*^Bc^*	52.5 ± 0.1*^Ac^*
*Rhizoctonia solani*	56.8 ± 0.1*^Bb^*	70.7 ± 0.1*^Ab^*
*Fusarium oxysporum*	36.1 ± 0.1*^Bc^*	50.1 ± 0.1*^Ac^*
*Botrytis cinerea*	67.3 ± 0.1*^Bab^*	85 ± 0.1*^Aa^*
*Valsa mali*	76.4 ± 0.1*^Ba^*	78.8 ± 0.1*^Aab^*
*Sclerotinia sclerotiorum*	74.0 ± 0.1*^Ba^*	84.1 ± 0.1*^Aa^*

Different lowercase letters in the same column indicate a significant difference in the antibacterial activity of lichesterinic acid against different pathogenic fungal strains (*p* < 0.05); different uppercase letters in the same row indicate a significant difference in the antibacterial activity of lichesterinic acid compared with fusaric acid against the same pathogenic fungal strain (*p* < 0.05).

### 3.3 Evaluation of the combined effects of lichesterinic acid with antibiotics

The active monomeric compound lichesterinic acid demonstrated an additive effect when combined with six different antibiotics against MRSA. In experiments involving *L. seeligeri*, lichesterinic acid exhibited synergistic effects when used in conjunction with piperacillin, oxacillin and chloramphenicol but had an additive effect when combined with other antibiotics (Table 3). This synergistic or additive interaction suggests that lichesterinic acid may enhance the antibacterial efficacy of antibiotics when used together.

**TABLE 3:** FICI values of lichesterinic acid combined with six antibiotics.

Strain	FICI					
	Vancomycin	Ciprofloxacin	Cefuroxime	oxacillin	Piperacillin	Chloramphenicol
MRSA	0.75 (+)	0.53 (+)	0.63 (+)	0.53 (+)	0.75 (+)	0.75 (+)
*Listeria seeligeri*	1.00 (+)	0.56 (+)	0.63 (+)	**0.5 (++)**	**0.5 (++)**	**0.5 (++)**

FICI ≤ 0.5 indicates a synergistic effect (++); 0.5 < FICI ≤ 1 indicates an additive effect (+); 1 < FICI ≤ 2 indicates an indifferent effect ( ± ); FICI > 2 indicates an antagonistic effect (−).

### 3.4 Safety evaluation of lichesterinic acid

**FIGURE 3:**
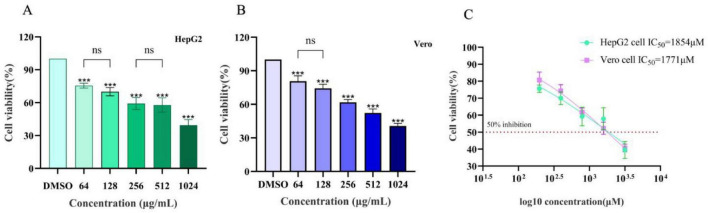
Assessment of the toxicity of lichesterinic acid on hepatic and renal cells. **(A)** HepG2 cells were treated with lichesterinic acid at 64, 128, 256, 512, and 1,024 μg/mL for 24 h, and cell viability was measured using the CCK-8 assay. **(B)** Vero cells were treated under the same conditions as in **(A)**. **(C)** IC^50^ calculated from the dose–response curves. Data are mean ± SD of four technical replicates in each of four independent experiments. Statistical significance was assessed by one-way ANOVA followed by Tukey’s multiple comparisons test. Asterisks denote significant differences compared with the control group (***p < 0.001); “ns” indicates no significant difference (*p* > 0.05).

To evaluate the cytotoxicity of lichesterinic acid, the human liver cancer cell line HepG2 and African green monkey kidney cell line Vero were selected for treatment at different concentrations (64, 128, 256, 512, and 1,024 μg/mL), and cell viability was measured. The results indicated that lichesterinic acid exhibited dose-dependent toxicity in both cell lines, with a gradual decline in cell viability as the concentration increased (Figures 3A,B). At the highest tested concentration (1,024 μg/mL), the viability of the HepG2 and Vero cells decreased to 39.47 and 40.67, respectively. Across other concentration ranges, the cell viability remained between 50 and 80%, indicating that the compound has overall low cytotoxicity. Further calculations revealed that the half-maximal inhibitory concentrations (IC^50^) for HepG2 and Vero cells were 1,854 and 1,771 μM, respectively (Figure 3C), suggesting that lichesterinic acid has good safety within the effective concentration range.

**FIGURE 4:**
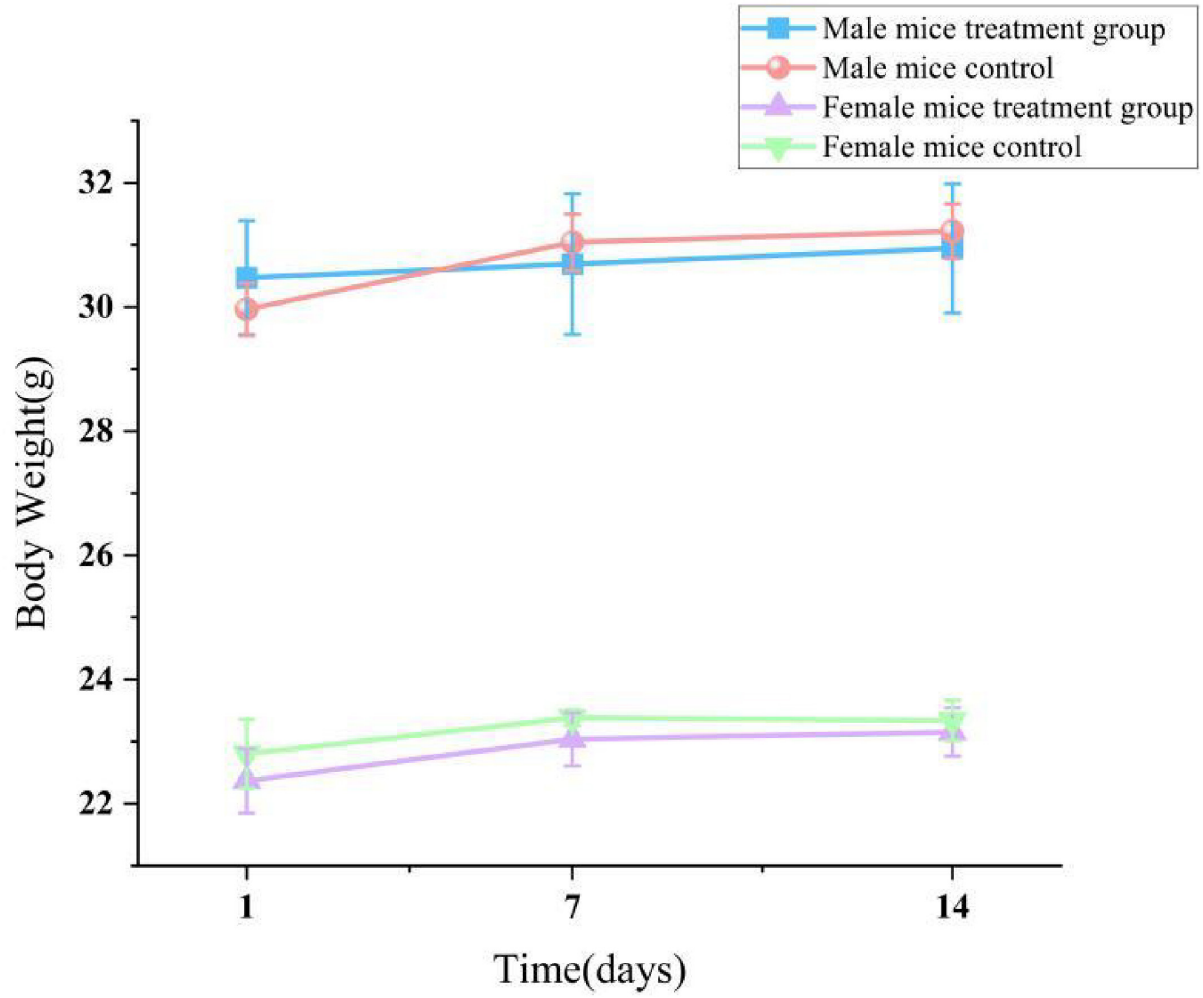
Effect of lichesterinic acid on body weight in mice. Body weights of mice (*n* = 20; 10 males and 10 females) were recorded on days 1, 7 and 14 following a single oral gavage of lichesterinic acid (5,000 mg/kg). The vehicle control group received an equivalent volume of corn oil. Data are presented as mean ± SD for each group. Statistical analysis was performed using one-way ANOVA followed by Dunnett’s multiple comparisons test.

In the acute toxicity experiment, after administering lichesterinic acid at a dosage of 5,000 mg/kg to the mice by gavage, transient toxic effects, including lethargy and drowsiness, were observed within the first 24 h; these symptoms resolved spontaneously after 24 h. During the subsequent 14-day observation period, the behavioral state, feeding, breathing, and other physiological activities of all the experimental mice remained normal, with no clinical symptoms, such as abnormal discharges, seizures, or wheezing, observed. No animal fatalities or other significant toxic reactions were noted. During the 14-day acute oral toxicity study, considering the inherent differences in body weight and physiological structure between male and female mice of the same age, male mice consistently presented significantly greater body weights than females did (*p* < 0.05). This study focused more on comparisons between different dosage groups within the same sex. The experimental results indicated that all the treatment groups and the control group presented stable increases in body weight during the experiment (Figure 4), with no significant differences in weight gain observed between any of the dosage groups and the control group (*p* > 0.05). These results suggest that lichesterinic acid had no significant adverse effects on the weight gain of the mice at the tested dosages.

**FIGURE 5:**
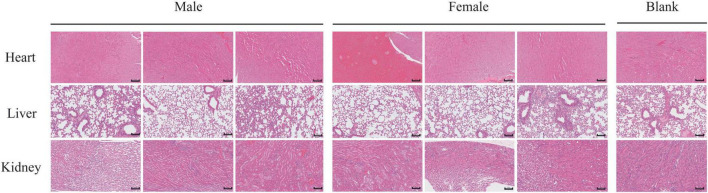
Histological examination of lung, kidney, and heart tissue sections stained with HE. Representative tissue sections of the lung, kidney, and heart were collected from mice stained with hematoxylin and eosin (H&E). Sections were examined under light microscopy to assess tissue morphology. Scale bars = 100 μm.

Necropsy revealed no gross pathological lesions in any organ. The heart, liver, spleen, kidneys, lungs, stomach, and gonads (testes or ovaries) were excised and weighed to calculate organ-to-body weight ratios. As shown in Table 4, the pulmonary index in treated female mice were significantly elevated relative to the control group (*p* < 0.05). No other organs exhibited dose-dependent alterations (*p* > 0.05). As shown in Table 5, high-dose lichesterinic acid significantly elevated total white blood cell (WBC) counts in male mice compared with vehicle controls (*p* < 0.05). In contrast, red blood cell counts, hemoglobin concentration, platelet counts, and leukocyte differentials (neutrophils, lymphocytes, and monocytes) remained unchanged (*p* > 0.05). Biochemical analyses (Table 6) revealed that albumin (ALB) levels in the male mice were significantly lower than in the control group (*p* < 0.05), whereas all other measured parameters did not differ significantly (*p* > 0.05). Histopathological examination at necropsy showed only minimal desquamation of the bronchial epithelium (consistent with physiological turnover), mild renal congestion, and no cardiac abnormalities. Overall, no treatment-related pathological changes were observed. At termination, gross necropsy and subsequent histopathological evaluation revealed only minimal desquamation of the bronchial epithelium (indistinguishable from normal physiological turnover), mild renal congestion, and an absence of cardiac lesions. No treatment-related pathological alterations were detected (Figure 5).

**TABLE 4:** Effects of lichesterinic acid on organ indices in mice.

Sex	Organ	Control 0 mg/kg (Mean ± SD)	High dose 5,000 mg/kg (Mean ± SD)	*P*-value
Male	Heart	0.83 ± 0.05*^a^*	0.70 ± 0.05*^a^*	0.340
	Liver	5.87 ± 0.04*^a^*	5.71 ± 0.09*^a^*	0.404
	Spleen	0.40 ± 0.01*^a^*	0.36 ± 0.01*^a^*	0.268
	Lung	0.62 ± 0.02*^a^*	0.67 ± 0.02*^a^*	0.349
	Kidney	1.24 ± 0.01*^a^*	1.20 ± 0.02*^a^*	0.539
	Stomach	0.78 ± 0.03*^a^*	0.75 ± 0.03*^a^*	0.711
	Testis	0.63 ± 0.02*^a^*	0.68 ± 0.02*^a^*	0.269
Female	Heart	0.60 ± 0.01*^a^*	0.77 ± 0.04*^a^*	0.182
	Liver	4.92 ± 0.03*^a^*	4.87 ± 0.06*^a^*	0.803
	Spleen	0.34 ± 0.01*^a^*	0.35 ± 0.01*^a^*	0.578
	Lung	0.75 ± 0.01*^b^*	0.85 ± 0.01*^a^*	**0.038**
	Kidney	1.15 ± 0.02*^a^*	1.14 ± 0.01*^a^*	0.894
	Stomach	0.85 ± 0.02*^a^*	0.85 ± 0.02*^a^*	0.939
	Ovary	0.08 ± 0.00*^a^*	0.09 ± 0.00*^a^*	0.192

All data are expressed as the means ± standard deviations (*n* = 6). Different lowercase letters (a, b) indicate significant differences between the control and treatment groups (*p* < 0.05). Organ weight index = (organ weight × 100)/body weight.

**TABLE 5:** Effects of lichesterinic acid on hematological parameters in mice.

Sex	Items	Control 0 mg/kg (mean ± SD)	High dose 5,000 mg/kg (mean ± SD)	*P*-value
Male	Hgb (g/L)	14.0 ± 1.2*^a^*	13.2 ± 0.6*^a^*	0.367
	RBC (10^9^/L)	10.2 ± 0.1*^a^*	9.8 ± 0.4*^a^*	0.377
	WBC (10^9^/L)	6.0 ± 0.6*^b^*	8.5 ± 1.2*^a^*	**0.031**
	PLT (10^9^/L)	2095.3 ± 940.5*^a^*	2148.7 ± 676.9*^a^*	0.94
	NEUT%	15.7 ± 3.9*^a^*	15.0 ± 3.4*^a^*	0.843
	LYMPH%	83.6 ± 6.4*^a^*	80.5 ± 3.6*^a^*	0.5
	MONO%	1.2 ± 0.1*^a^*	1.0 ± 0.3*^a^*	0.44
Female	Hgb (g/L)	13.6 ± 0.7*^a^*	12.8 ± 0.4*^a^*	0.177
	RBC (10^9^/L)	9.8 ± 0.5*^a^*	9.4 ± 0.4*^a^*	0.318
	WBC (10^9^/L)	5.2 ± 1.3*^a^*	7.7 ± 1.3*^a^*	0.072
	PLT (10^9^/L)	1514.3 ± 251.0*^a^*	1601.3 ± 185.4*^a^*	0.654
	NEUT%	12.7 ± 1.2*^a^*	14.7 ± 3.0*^a^*	0.339
	LYMPH%	84.5 ± 1.2*^a^*	81.4 ± 3.8*^a^*	0.259
	MONO%	1.2 ± 0.1*^a^*	1.0 ± 0.3*^a^*	0.374
	Hgb (g/L)	13.6 ± 0.7*^a^*	12.8 ± 0.4*^a^*	0.177

Hgb, Hemoglobin; RBC, Red Blood Cell Count; WBC, White Blood Cell Count; PLT, Platelet Count; NEUT, Neutrophils; LYMPH, Lymphocytes; MONO, Monocytes. Different lowercase letters (a,b) indicate significant differences in the hematological indicators among different between the control and treatment groups (*p* < 0.05).

**TABLE 6:** Effects of lichesterinic acid on biochemical indicators in mice.

Sex	Items	Control 0 mg/kg (mean ± SD)	High dose 5,000 mg/kg (mean ± SD)	*P*-value
Male	ALB (g/L)	32.5 ± 0.4*^a^*	30.0 ± 1.0*^b^*	**0.014**
		ALT (U/L)	46.3 ± 9.1*^a^*	42.0 ± 9.8*^a^*	0.605
		AST (U/L)	199.0 ± 13.7*^a^*	171.7 ± 41.5*^a^*	0.340
		ALP (U/L)	14.7 ± 2.5*^a^*	11.7 ± 2.5*^a^*	0.218
		BUN (mmol/L)	10.7 ± 0.9*^a^*	9.5 ± 0.9*^a^*	0.200
		CREA (mmol/L)	35.0 ± 5.2*^a^*	23.7 ± 9.0*^a^*	0.131
		TG (mmol/L)	1.8 ± 0.3*^a^*	1.8 ± 0.3*^a^*	0.970
		CHOL (mmol/L)	2.6 ± 0.1*^a^*	2.7 ± 0.3*^a^*	0.842
Female	ALB (g/L)	30.7 ± 5.1*^a^*	28.4 ± 6.6*^a^*	0.656
		ALT (U/L)	30.0 ± 12.2*^a^*	27.3 ± 9.8*^a^*	0.782
		AST (U/L)	142.3 ± 8.5*^a^*	156.0 ± 59.6*^a^*	0.714
		ALP (U/L)	13.7 ± 2.1*^a^*	10.7 ± 2.3*^a^*	0.170
		BUN (mmol/L)	10.8 ± 1.7*^a^*	9.4 ± 1.7*^a^*	0.365
		CREA (mmol/L)	28.7 ± 8.6*^a^*	17.7 ± 0.6*^a^*	0.092
		TG (mmol/L)	0.86 ± 0.3*^a^*	0.96 ± 0.4*^a^*	0.759
		CHOL (mmol/L)	1.9 ± 0.3*^a^*	1.7 ± 0.3*^a^*	0.594

ALB, Albumin; ALT, alanine aminotransferase; AST, aspartate aminotransferase; ALP, alkaline phosphatase; BUN, blood urea nitrogen; CREA, creatinine; TG, triglycerides; CHOL, total cholesterol. Different lowercase letters (a,b) indicate significant differences in the biochemical parameters between the control and treatment groups (*p* < 0.05).

### 3.5 Molecular docking of lichesterinic acid and *S. aureus*-resistant proteins

**FIGURE 6:**
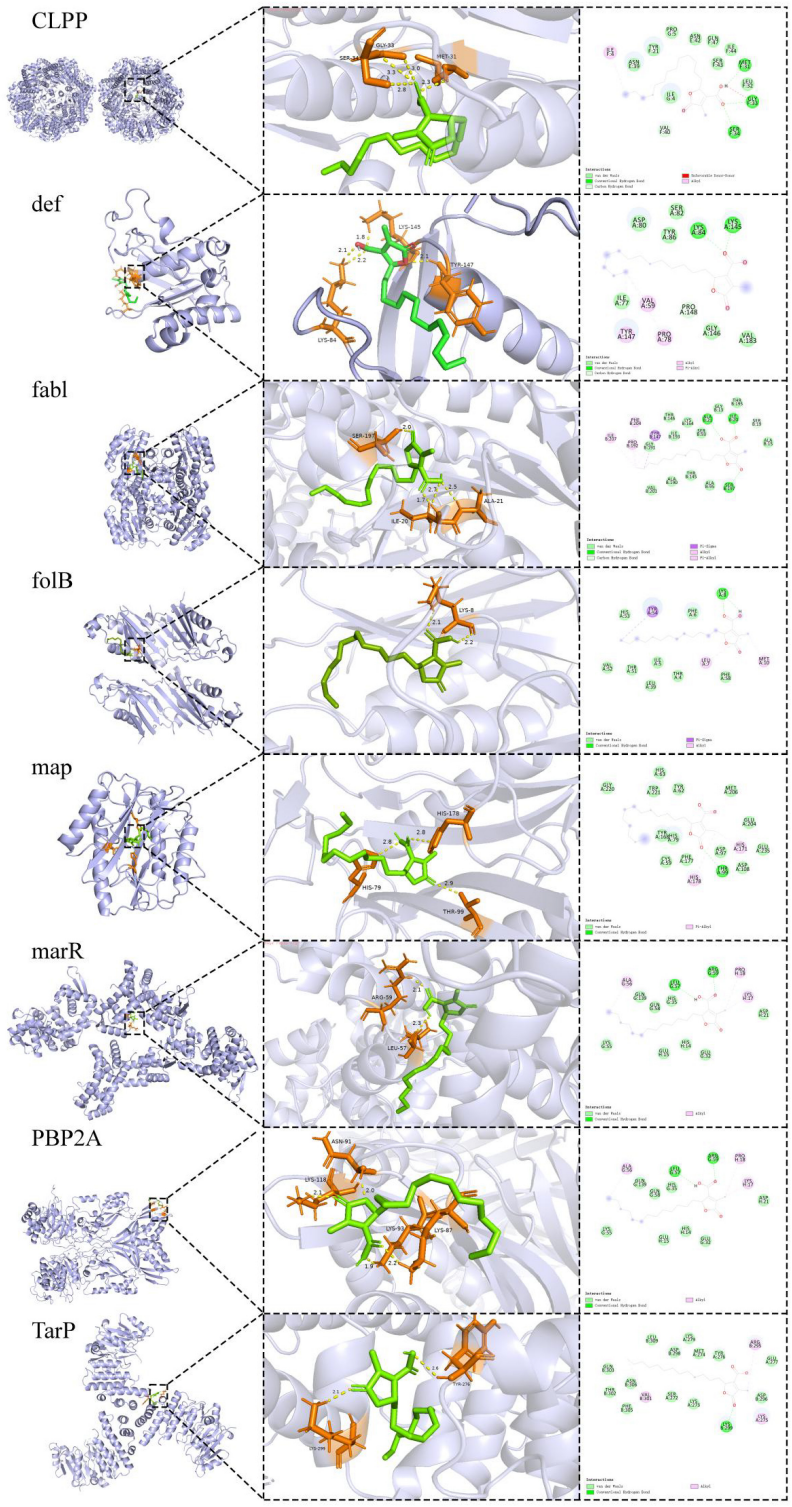
Molecular docking of lichens acid and *S. aureus*-resistant proteins. Predicted binding poses of lichesterinic acid with the indicated target proteins. 3D visualizations showing lichesterinic acid (stick model) in the binding pocket of the proteins (ribbon model). 2D diagrams illustrating the detailed ligand-protein interactions.

The combined use of free energy change (ΔG) and conformational stability (RMSD) may serve as a practical and efficient criterion in the initial screening of molecular docking (Han et al., 2023; Ni et al., 2024). The comparative docking results are summarized in Table 7 and illustrated in Figure 6. TarP showed the most favorable predicted binding energy (ΔG = −7.14 kcal/mol), but a relatively high RMSD (2.948 Å), suggesting that although the energy score is good the top pose may be conformationally less stable. Def exhibited a comparably low binding energy (−7.06 kcal/mol) and a moderate RMSD value (2.034 Å). It formed multiple stabilizing interactions, including two conventional hydrogen bonds (LYS A:84 and LYS A:145), as well as van der Waals and alkyl contacts, indicating favorable binding stability and specificity. PBP2a exhibits a stable docking pose (RMSD = 1.601 Å) with moderate binding affinity (ΔG = −6.30 kcal/mol); although no hydrogen bonds were observed, extensive van der Waals contacts contribute to a reasonable interaction surface. Map exhibits a poorer binding energy (ΔG = −5.80 kcal/mol) but the minimal RMSD (1.21 Å), indicating its docked conformation possesses high reproducibility and remains worthy of consideration. FabI and CLPP return relatively high RMSD values (>3.2 Å), signifying lower pose stability despite moderate-to-low energy scores. FolB forms a critical hydrogen bond with LYS A:8, potentially enhancing binding specificity.

**TABLE 7:** Molecular docking results of lichesterinic acid with eight bacterial target proteins.

Target	Binding energy (kcal/mol)	RMSD (Å)	Key interaction types	Key residues involved
Def (6JFQ)	−7.06	2.034	vdW, H-bonds, Alkyl	ASP A:80, SER A:82, TYR A:86, LYS A:84/145 (H-bonds), VAL A:59, ILE A:77
TarP (6H2N)	−7.14	2.948	vdW	LYS A:279, MET A:274, TYR A:276, GLN B:303, PHE B:305
PBP2a (6H5O)	−6.3	1.601	vdW	LYS B:118, LYS B:90, LYS B:92, TRP B:123, ASN B:91
FabI (4ALL)	−6.54	3.226	vdW	THR B:146, LYS B:164, ALA B:21, SER B:197
CLPP (3V5I)	−6.5	3.923	vdW	PRO G:5, ASN E:42, GLN F:47, TYR F:21, ILE F:44
Map (1C21)	−5.8	1.21	vdW	HIS A:63, TRP A:221, TYR A:62, PHE A:177, CYS A:59
folB (2NM2)	−5.8	2.039	vdW, H-bond	PHE A:6, LYS A:8 (H-bond), MET A:10
marR (4LD5)	−5.9	2.55	vdW	ALA G:56, GLN G:58/139, LEU G:57, HIS G:35

## 4 Discussion

The world is facing an increasingly severe global health crisis: antibiotic resistance, making the search for and development of new antibacterial agents an urgent priority in the scientific community (Kaushik et al., 2024). Lichens are rich in various unique active substances and serve as important sources of new structurally diverse compounds (Ren et al., 2023). Our research team previously discovered that lichens growing in the moss plant zone of Cangshan exhibit good antibacterial activity against MRSA, but no studies have been conducted on the active substances underlying this antibacterial activity, their mechanisms of action, or their potential for drug development.

To further explore the antibacterial constituents of these lichens, the crude extracts were first fractionated by silica gel column chromatography. During this process, dichloromethane-methanol and petroleum ether-ethyl acetate systems were used as elution solvents, and a gradient of 1:0–0:1 (v/v) was used to elute the metabolites. Surprisingly, nearly 80% of the isolated fractions exhibited significant antibacterial activity against MRSA. Subsequent thin-layer chromatography analysis revealed that certain fractions not only showed outstanding activity but also allowed for easy precipitation of the target substances, greatly facilitating further isolation and enrichment. On the basis of these characteristics, gel column chromatography and preparative thin-layer chromatography were successfully employed to isolate and purify the target monomer compound—lichesterinic acid.

Lichesterinic acid belongs to the class of fatty acids, and studies have shown that high fatty acid and ester contents in lichens demonstrate notable antimicrobial properties. This study determined the MIC of lichesterinic acid against 16 pathogenic bacteria. The results showed that lichesterinic acid exhibited the strongest antibacterial activity against *S. aureus* and *L. seeligeri*, both with MIC values of 64 μg/mL. *S. aureus* is a significant pathogen responsible for various infections, including skin infections, pneumonia, and sepsis (Cheung et al., 2021), whereas *Listeria monocytogenes* is a highly resilient foodborne pathogen that can grow at refrigerator temperatures, posing a considerable challenge to food preservation, with infections potentially leading to severe diseases such as meningitis and sepsis (Koopmans et al., 2023). Therefore, the strong inhibitory activity of lichesterinic acid against these pathogens is of vital clinical and public health significance. Extracts containing protolichesterinic acid (tautomers of lichesterinic acid) from *Cetraria aculeata* have also been shown to be effective against pathogenic bacteria such as *S. aureus* and *Listeria* (Türk et al., 2003), further supporting the potential of such compounds as food preservatives. Additionally, lichesterinic acid exhibits antibacterial activity against MRSA, *S. Paratyphi* A and B, *P. fluorescens*, *S. epidermidis*, and *Ralstonia solanacearum*, indicating its broad-spectrum antibacterial potential. Fatty acid compounds such as lichesterinic acid generally have better inhibitory effects on gram-positive bacteria (such as *Staphylococcus*, *Listeria*, and *Bacillus*) than on gram-negative bacteria (such as *Escherichia coli* and *Pseudomonas aeruginosa*) (Tian et al., 2025). This strong selectivity hints at the characteristics of its mechanism of action. Gram-negative bacteria possess an outer membrane composed of lipopolysaccharides, which serve as a barrier that many lipophilic compounds find difficult to breach (Adenubi et al., 2022). The difficulty of molecules such as lichesterinic acid penetrating this outer membrane may explain their generally lower activity against gram-negative bacteria. This observation further suggests that their action targets are likely located in the cytoplasmic membrane or within the cells themselves.

Furthermore, to clarify its *in vitro* antifungal activity against plant pathogens, nine common plant pathogenic fungi were selected for testing. Notably, *B. cinerea*, *Fusarium oxysporum* f.sp. *niveum*, and *Fusarium graminearum* have been recognized as among the 10 most important fungal pathogens in the field of molecular plant pathology (Dean et al., 2012). The results from confrontation assays revealed that this compound has good inhibitory effects on *S. sclerotiorum*, *V. mali*, *B. cinerea*, and *C. orbiculare*, with inhibition rates exceeding 60%. These pathogenic fungi have a wide host range and can cause significant yield losses in various crops, leading to substantial economic impacts while also demonstrating resistance to traditional agricultural fungicides (Imran et al., 2021; Outwater et al., 2020; Zhang et al., 2022). Compared with conventional chemical fungicides, lichesterinic acid is natural in nature, has good environmental degradability, and has the potential to be developed as a “green antibacterial agent” (Qi et al., 2023). As an independent antibacterial drug, the significant activity of lichesterinic acid (MIC of 64 μg/mL) renders it a candidate for a new class of antibiotics.

In the combined antimicrobial test, lichenic acid reduced the MIC of the 6 antibiotics tested and exhibited synergistic effects in the form of FICI values ranging from 0.5 to 1.0. Bellio et al. (2015) similarly proposed that lichen-derived metabolites may potentiate antibiotic activity by increasing bacterial membrane permeability, inhibiting efflux pumps, or modulating resistance determinants. Taken together, these findings indicate that lichesterinic acid may act as an antibiotic synergist, enhancing the antibacterial efficacy of existing drugs and potentiating current treatments, although its precise molecular mechanism remains to be elucidated.

To further clarify the application potential of the active monomer compound, the cytotoxicity of lichesterinic acid to HepG2 and Vero cells at different concentrations was assessed. The results indicated that as the concentration of lichesterinic acid increased, the cell viability gradually decreased; however, its cytotoxicity remained relatively low. At the highest tested concentration (1,024 μg/mL), the cell viability decreased to less than 50%, whereas at other concentrations, the viability ranged between 50 and 80%. The calculated IC_50_ values for lichesterinic acid against HepG2 and Vero cells were 1,854 and 1,771 μM, respectively. These data indicate that lichesterinic acid is weakly toxic to both cell lines, as cell viability was not completely lost even at high concentrations. This characteristic of low toxicity may be related to the chemical structure of lichesterinic acid or its specific mechanism of action, suggesting that it may exert effects by modulating cellular metabolism or physiological functions rather than directly inducing cell death. Compared with other lichen-derived extracts, such as *Usnea antarctica* and *Usnea aurantiacoatra*, which presented IC_50_ values against Vero cells of 169.64 and 270.82 μg/mL, respectively (Londoñe-Bailon et al., 2019), lichesterinic acid showed lower cytotoxicity. These results indicate that lichesterinic acid causes minimal cellular damage at low concentrations, demonstrating better safety and potential medicinal value.

Currently, literature regarding the *in vivo* toxicological data of purified lichesterinic acid is extremely scarce, with almost no reports on its acute toxicity (LD_50_) or subchronic toxicity studies in animal models (European Medicines Agency [EMA], 2014). This study involved a single oral administration of 5,000 mg/kg of lichesterinic acid to mice, with a comprehensive assessment of its acute toxicity over a 14-day observation period. Behavioral observations indicated that after administration, the mice displayed drowsiness, reduced activity, and clustering behavior, which spontaneously normalized after 4 h, with no further abnormal behaviors, deaths, or other typical toxic manifestations observed. Throughout the experiment, all the mice exhibited a stable weight increase trend, with no significant differences between the treatment and control groups (*p* > 0.05), further indicating good tolerability at this dosage. Organ index analysis did not reveal significant organ toxicity; only the lung index of the female treatment group was significantly greater than that of the control group (*p* < 0.05), although histological sections revealed only slight shedding of the bronchial mucosa without reaching the standard for lung damage. The heart index of the male treatment group was slightly lower than that of the control group (*p* < 0.05), but myocardial enzymatic indicators and histological examinations revealed no abnormalities, potentially attributable to individual differences. Serum biochemical indicators revealed that creatinine (CREA) levels in female mice significantly decreased (*p* < 0.05), but histology indicated only congestion, which did not reach the standard for kidney damage. In summary, a single oral dose of 5,000 mg/kg lichesterinic acid did not produce significant systemic toxicity in male or female mice, with stable weights, organ indices, and hematological and serum biochemical indicators, resulting in only minor changes in some instances. Based on the acute toxicity classification, lichesterinic acid has an LD_50_ of > > 5,000 mg/kg⋅bw, categorizing it as a nontoxic substance (Liu et al., 2023). Although natural products such as lichesterinic acid demonstrate marked antibacterial potency at low concentrations while exhibiting minimal cytotoxicity even at elevated doses (Kavitha et al., 2023), comprehensive subacute and chronic toxicological evaluations are still required to substantiate these preliminary safety conclusions.

In molecular docking studies, the binding energy and RMSD are widely used to evaluate the binding affinity and conformational stability between ligands and targets. Generally, lower binding energy (<−5 kcal/mol) and RMSD values (<2 Å) indicate closer binding between the ligand and the receptor and greater stability of the conformation (Liu et al., 2024). Docking results indicate that TarP exhibits a favorable predicted binding energy with lichesterinic acid, suggesting substantial thermodynamic propensity for interaction. TarP is a key enzyme in gram-positive bacteria that synthesizes wall teichoic acid (WTA), which is crucial for cell wall structural stability and virulence expression and is particularly critical in MRSA (Gerlach et al., 2022). TarP-mediated modification of WTA promotes evasion of host immune recognition, thereby enhancing bacterial pathogenicity. However, the TarP and lichesterinic acid exhibits a relatively high RMSD (2.948 Å) and lacks stabilizing complementary interactions, suggesting that binding may involve conformational strain. However, the TarP–ligand complex shows a relatively high RMSD (2.948 Å) and lacks an extensive network of stabilizing complementary interactions, which may reflect conformational strain during binding and reduce the likelihood of a well-defined, stable complex. Lichesterinic acid has lower binding energy and RMSD values at the active site of def, and the interactions between this ligand and def are diverse, including van der Waals forces, carbon–hydrogen bonds, alkyl interactions, and conventional hydrogen bonds, particularly forming two hydrogen bonds between LYS A:84 and LYS A:145, which may further increase the binding specificity and affinity, suggesting the potential to form stable and discernible binding conformations. Def is a metal enzyme widely present in bacteria that is involved in the removal of N-terminal formyl groups from nascent peptide chains, playing a critical role in posttranslational modifications of proteins (Kirschner et al., 2024). Owing to its high degree of conservation and essential nature in bacteria, lichesterinic acid may disrupt fundamental life processes in bacteria by inhibiting def, demonstrating broad-spectrum antibacterial potential. PBP2a is a core protein that mediates the resistance of MRSA to β-lactam antibiotics, is involved in cell wall synthesis (Ravi et al., 2019), and serves as an important target for penicillin antibiotics (Kalalo et al., 2020). In this study, lichesterinic acid demonstrated a lower RMSD and acceptable binding energy when combined with PBP2a, despite the absence of hydrogen bonds, achieving stable binding through van der Waals interactions. These findings suggest that the ligand may effectively interfere with MRSA resistance mechanisms by inhibiting PBP2a activity (Anwer, 2024). This mechanism may not only independently exert antibacterial effects but also potentially synergize with conventional β-lactam antibiotics, restoring sensitivity to MRSA, which has significant clinical implications. Moreover, molecular docking results identify Map and FolB as additional putative targets of lichesterinic acid.

Overall, lichesterinic acid can bind to various key target proteins, exhibiting a potential multitarget antibacterial mechanism, likely interfering with multiple bacterial survival pathways, including protein processing, cell wall synthesis, and immune evasion. Research indicates that strategies employing single-target combination therapy or developing multitarget antibacterial agents are effective in curbing the development of bacterial resistance (Baym et al., 2016). Therefore, future research could incorporate targeted proteomics techniques to further analyze the expression changes and interaction mechanisms of these potential antibacterial targets, providing theoretical foundations and practical guidance for the development of novel MRSA drugs.

## 5 Conclusion

This study aimed to investigate the antibacterial activity of lichesterinic acid from the Bryophyte belt in the snowy area of the Cangshan Plateau against MRSA and its potential for drug development. Using MRSA as the indicator strain, this research comprehensively employs techniques in natural product chemistry and pharmacology to identify lichesterinic acid as the antibacterial monomer compound in lichens, with a content of 0.32% in the crude extract, a molecular formula of C_19_HC_32_OC_4_, and a molecular weight of 324.455. Lichesterinic acid has a broad spectrum of antibacterial effects, particularly exhibiting good inhibitory effects against gram-positive bacteria, such as *S. aureus*, MRSA, and *Listeria monocytogenes*. Additionally, in the antifungal domain, lichesterinic acid effectively inhibits *B. cinerea*, black rot of apples, and *S. sclerotiorum*. Lichesterinic acid also has potential clinical application value through synergistic effects with antibiotics, providing new strategies for antibacterial therapy. In terms of safety, the results from cytotoxicity assays and acute toxicity experiments in animals indicate that lichesterinic acid is nontoxic and has good biosafety, which establishes an important foundation for its further development and application. Molecular docking studies revealed that Def (peptidyl deformylase) is a significant target of action for lichesterinic acid, offering critical clues for further exploration of its mechanisms of action. In summary, this research not only provides a scientific basis for the further development and application of lichesterinic acid but also opens new avenues for research on natural products with antibacterial activity. Moving forward, a systematic pipeline will be pursued that integrates *in vitro* biochemical/biophysical binding and inhibition assays, microbiological efficacy plus synergy/resistance profiling, cytotoxicity and PK/PD studies, and single-agent as well as combination efficacy and safety/toxicity evaluations in animal infection models. This will enable us to elucidate and validate the mechanism of action and clinical translation potential of lichesterinic acid.

## Data Availability

The original contributions presented in this study are included in this article/Supplementary material, further inquiries can be directed to the corresponding authors.

## Footnotes

1
https://www.lilab-ecust.cn/pharmmapper/index.html

2
https://www.rcsb.org/
