# Supplementary material for: Unearthing anti-MRSA agents from alpine lichens: discovery and characterization of bioactive compounds in Cetraria islandica from the snowy Cangshan region
Source: Front Microbiol. 2025 Nov 27;16:1688435. doi: 10.3389/fmicb.2025.1688435 (PMC12695739; doi:10.3389/fmicb.2025.1688435)
Supplement: Supplementary file 1 [file Table_1.docx]

Supplementary Material

# Experimental strains

| Strains | Strain Number | Source |
| --- | --- | --- |
| *Staphylococcus aureus* | ATCC 25923 | Basic Medical College of Dali University |
| *Escherichia coli* | CMCC(B)441027 |  |
| *Methicillin-resistant Staphylococcus aureus* | ATCC 43300 |  |
| *Staphylococcus epidermidis* | ATCC 12228 | Dali Prefecture Quality and Technical Supervision Comprehensive Testing Center |
| *Listeria monocytogenes* | CMCC 21633 |  |
| *Listeria seeligeri* | CICC 21671 |  |
| *Listeria lvanovii* | CICC 21663 |  |
| *Listeria innocua* | CICC 10417 |  |
| *Salmonella paratyphi-A* | BNCC 336664 |  |
| *Salmonella paratyphi-B* | BNCC103169 |  |
| *Bacillus cereus* | ATCC 6633 |  |
| *Klebsiella pneumoniae* | ATCC 4352 |  |
| *Pseudomonas aeruginosa* | ATCC 27853 | Beijing Beina Chuanglian Biotechnology Research Institute |
| *Solanacearum(Smith)Smith* | BNCC 335855 |  |
| *Agrobacterium tumefaciens* | BNCC 180854 |  |
| *Erwinia amylovora* | BNCC 137731 |  |
| *Pectobacterium carotovorum* | BNCC 139620 |  |
| *Candida albicans* | ATCC 10231 |  |
| *Saccharomyces* | BNCC 142268 |  |
| *Botrytis cinerea* | BNCC 119830 |  |
| *Rhizoctonia solani* | BNCC 356048 |  |
| *Fusarium oxysporum f.sp.niveum* | BNCC 225899 |  |
| *Sclerotinia sclerotiorum* | BNCC 122299 |  |
| *Colletotrichum orbiculare* | BNCC 226034 |  |
| *Fusarium graminearum* | BNCC 337560 |  |
| *Fusarium moniliforme* | BNCC 186247 |  |
| *Valsa mali* | BNCC 116391 |  |

# Main reagents

| Materials | Source |
| --- | --- |
| Ciprofloxacin (CIP), purity 98% | Shanghai Macklin Biochemical Co., Ltd |
| Vancomycin (VAN), purity ≥ 95% |  |
| Cefoxitin (FOX), purity 98% | Shanghai Aladdin Biochemical Technology Co., Ltd. |
| Tetracycline (TET), purity 96% |  |
| Chloramphenicol (CHL), purity 98% |  |
| Piperacillin (PIP), purity ≥ 95% |  |
| Oxacillin (OX), purity 95% | Beijing Solarbio Science & Technology Co., Ltd. |
| Levofloxacin tablets (Levofloxacin hydrochloride tablets) | Shandong Luoxin Pharmaceutical Group Co., Ltd. |
| 2,3,5-Triphenyltetrazolium chloride (TTC) | Xi'an Xinyu Chengzhi Trading Co., Ltd. |
| Sabouraud dextrose liquid medium | Guangdong Huankai Microbial Sci.&Tech.Co.,Ltd |
| Potato dextrose agar medium (PDA) |  |
| Nutrient Agar |  |
| Nutrient Broth |  |
| LB Broth |  |
| Tryptic Soy Agar (TSA) |  |
| Mueller–Hinton Agar (MH agar) |  |
| Mueller–Hinton Broth (MHB) |  |
| GF254 silica gel thin-layer chromatography plates | Qingdao Marine Chemical Factory |
| Dimethyl sulfoxide (DMSO) | Tianjin Combi Chemical Reagent Co., Ltd. |
| E.Z.N.A.™ Mag-Bind Soil DNA Kit (M5635‑02) | OMEGA |
| Qubit dsDNA HS Assay Kits (Q32854) | Thermo Fisher Scientific Inc.， |
| 2× Hieff® Robust PCR Master Mix (10105ES03) | Shanghai Yeasen Biological Technology Co., Ltd. |
| Hieff™ NGS DNA Selection Beads (12601ES56) |  |
| Silica gel powder (200–300 mesh) | Qingdao Marine Chemical Co., Ltd. |
| Methanol | Sichuan Xilong Scientific Co., Ltd. |
| Ethyl acetate, AR |  |
| Petroleum ether, AR (30–60 °C) |  |
| Dichloromethane, AR |  |
| 1‑Butanol, AR |  |
| Ethanol, AR |  |
| Chloroform, AR |  |
| n‑Hexane, AR |  |
